# Supplementary material for: Machine Learning Uncovers a Data-Driven Transcriptional Regulatory Network for the Crenarchaeal Thermoacidophile Sulfolobus acidocaldarius
Source: Front Microbiol. 2021 Oct 27;12:753521. doi: 10.3389/fmicb.2021.753521 (PMC8578740; doi:10.3389/fmicb.2021.753521)
Supplement: Supplementary file 7 [file Data_Sheet_7.PDF]

Supplementary Document

Main Manuscript: Machine learning uncovers a data-driven transcriptional regulatory network for the Crenarchaeal thermoacidophile Sulfolobus acidocaldarius

Supplementary Figure 1

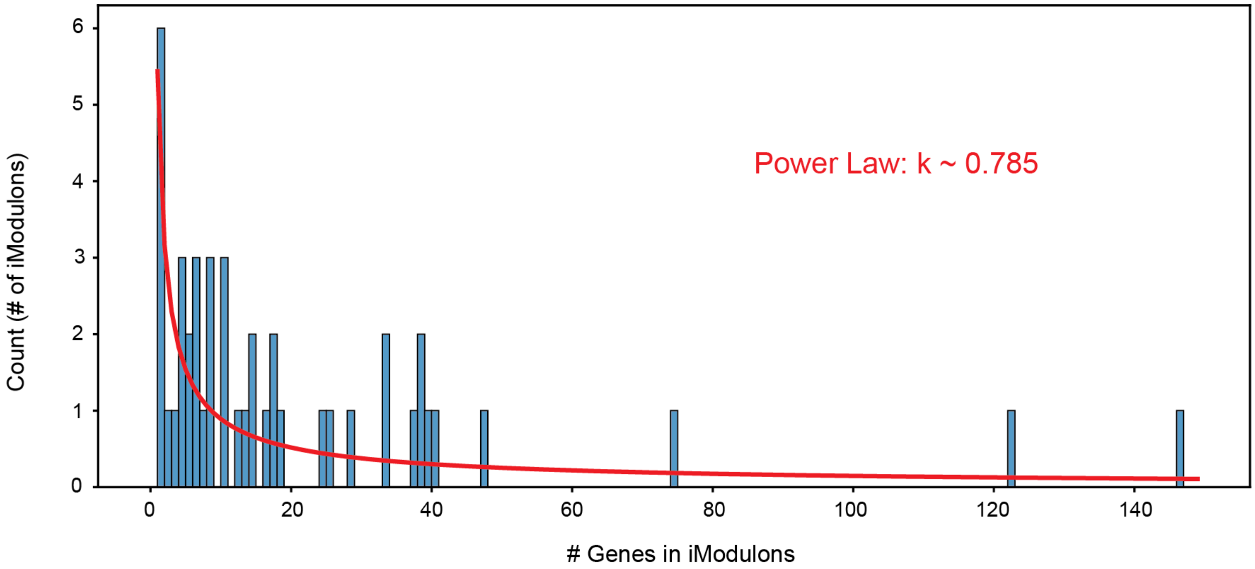

Supplementary Figure 1: Histogram of iModulons by gene set size (bin size of 1). Most iModulons contain below 20 genes, and all but 3 iModulons contain below 50 genes. Power Law formulation (with exponent parameter of ~0.785) overlaid this distribution in red.

Supplementary Figure 2

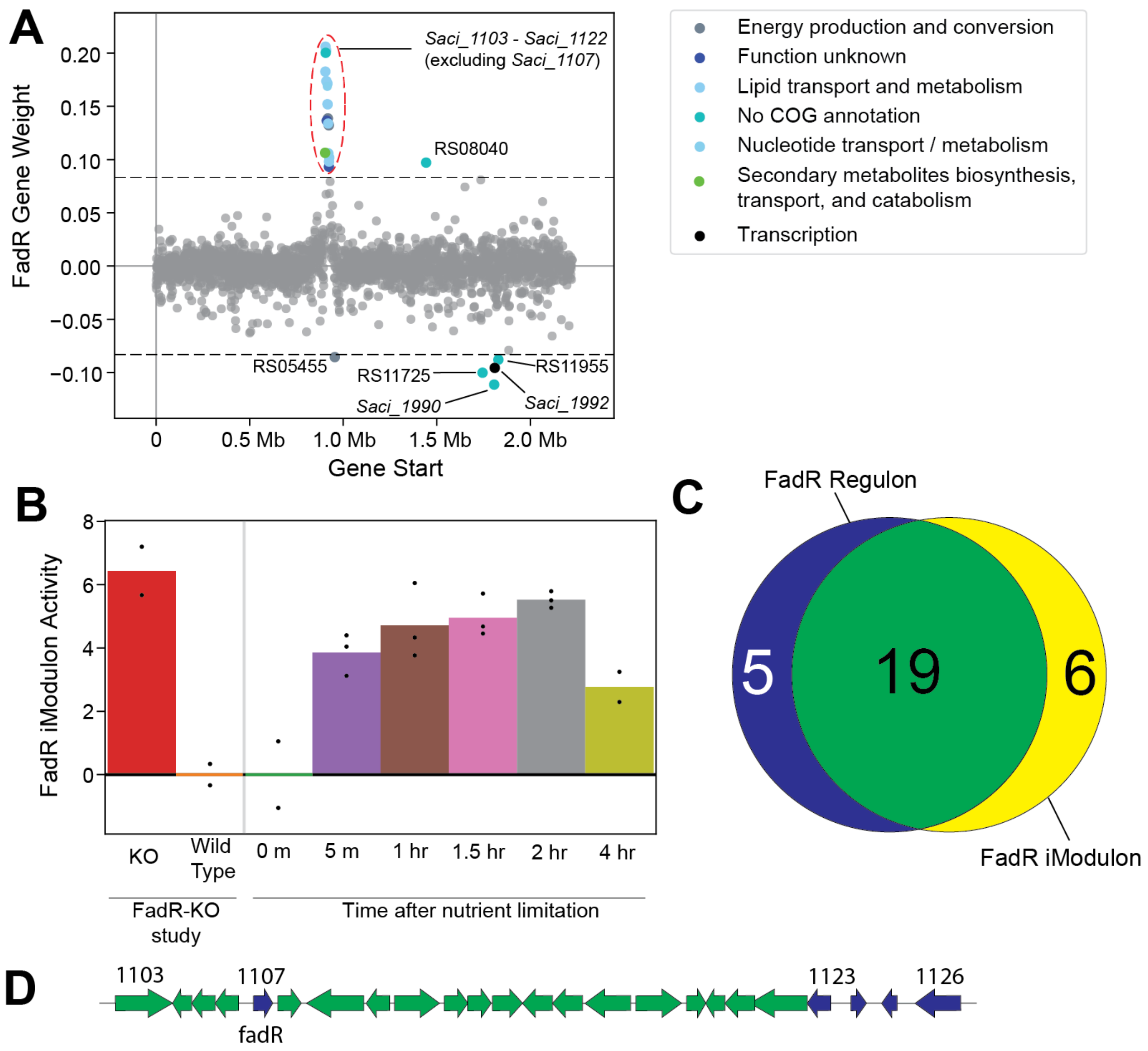

Supplementary Figure 2: Overview of the FadR iModulon. **a)** Scatter plots of the FadR iModulon gene weights. Genes are colored by COG categories. **b)** Barplot of FadR iModulon activity in 2 projects. **c)** Venn diagram comparing iModulon Recall with Regulon Recall. **d)** Gene map of FadR gene cluster in *S. acidocaldarius*. Green genes are in both iModulon and regulon, and blue genes are only in the regulon.

Supplementary File 1: A metadata file containing all RNA-seq datasets considered in this study, along with accession numbers (SRA and BioProject) alongside relevant PMID values for relevant manuscripts.

Supplementary File 2: A draft TRN (tsv and xlsx format) assembled from extensive literature review. Each gene-regulation interaction is mapped towards a PMID listing the reference where it was first reported.

Supplementary File 3: A curated genome annotation (tsv and xlsx format) file

Supplementary File 4: InterPro scan results (json and gff format) for uncharacterized genes in UV-tfb3 iModulon (*Saci_0951, Saci_1225, Saci_1270, Saci_1302,* and SACI_RS06010)

Supplementary File 5: InterPro scan results (json and gff format) for putative peptide-induced D-xylose transporters (*Saci_0324*, *Saci_0675*, and *Saci_2095*)

Supplementary File 6: Excel sheet (xlsx format) of iModulon tables with gene member sets, gene names, old locus tags, and arCOG values. Known and putative TFs are highlighted in light red.
